# Prevalence of arterial hypertension among Brazilian adolescents: systematic review and meta-analysis

**DOI:** 10.1186/1471-2458-13-833

**Published:** 2013-09-11

**Authors:** Erika Silva Magliano, Luciane Gaspar Guedes, Evandro Silva Freire Coutinho, Katia Vergetti Bloch

**Affiliations:** 1 Instituto de Estudos em Saúde Coletiva, Universidade Federal do Rio de Janeiro, Rio de Janeiro, Brazil; 2 Avenida Horácio Macedo, S/N – Próximo a Prefeitura Universitária, Ilha do Fundão, Cidade Universitária, CEP 21941-598 Rio de Janeiro - RJ, Brazil; 3 Escola Nacional de Saúde Pública, Fundação Oswaldo Cruz, Endereço: Rua Leopoldo Bulhões 1480, Manguinhos, CEP 21041-210 Rio de Janeiro- RJ, Brazil

**Keywords:** Hypertension, Teenager, Brazil, Systematic review, Meta-analysis

## Abstract

**Background:**

Cardiovascular diseases are the leading cause of death in the world and are responsible for a high number of disability-adjusted life years. Elevated blood pressure is an independent, linear and continuous risk factor for cardiovascular disease and has also been reported in the young population. Brazil is a country of continental dimensions, and is very heterogeneous with respect to socioeconomic and cultural aspects. Brazilian studies on the subject of hypertension in adolescence are not nationally representative, and this provides a rationale for the conduction of a meta-analysis to assess the magnitude of the problem in the country.

**Methods:**

Hypertension studies in adolescents published from 1990 to September 2010 were searched in six electronic databases. Forest plots of the prevalence of hypertension were built for the overall population and by gender. Heterogeneity was assessed using I^2^ statistics. Meta-regression models were adjusted in order to identify possible sources of heterogeneity.

**Results:**

Of 3,631 articles initially identified, 17 were considered eligible for systematic review. The pooled prevalence of hypertension, estimated through random effects models, was 8.12% (95% CI 6.24 - 10.52) for the total population. Overall, prevalence was higher in males, 8.75% (95% CI 5.81 - 12.96) than females, 6.31%, (95% CI 4.41 - 8.96). Several variables were investigated in the heterogeneity analysis: region of the study, sample size, age and method of blood pressure measurement. The only variables that partially and inconsistently explained the observed heterogeneity (I^2^ = 95.3%) were the region of the country where the study was conducted and sample.

**Conclusions:**

There was a large variation in hypertension prevalence and in the methods used for its evaluation throughout studies with Brazilian adolescents, indicating the need for standardized procedures and validated methods for hypertension measurement. Despite the large observed heterogeneity, and the small number of studies in some regions of Brazil, the pooled prevalence found in both males and females shows that systemic arterial hypertension should be monitored in the population aged 10–20 years and that specific measures are required to prevent and control the disease, as well as its risk factors. Studies that compare regional heterogeneities may contribute to the knowledge of factors associated with increased blood pressure among adolescents.

## Background

Throughout the world, cardiovascular diseases are the leading cause of death in the overall population and are also responsible for a high number of disability-adjusted life years (DALY). A study using DALY showed that in Brazil there is a predominance of noncommunicable diseases, accounting for 66.3% of the disease burden [1].

A review of 13 studies carried out in Brazil after 1990 showed that the prevalence of hypertension ranged from 24.8% to 44.4% [2].

There is an increase in diseases associated with excess weight, leading to cardiovascular problems in adolescents, such as elevated blood pressure [3,4]. The Family Expenditure Survey (Pesquisa de Orçamento Familiar) showed that the diet of Brazilian citizens, including adolescents, has foods with reduced nutrients and high calorie content [5]. Furthermore, data from the National Health Survey of Schoolchildren (Pesquisa Nacional de Saúde do Escolar) reported low levels of physical activity and sedentary habits among adolescents [6].

In children and adolescents, hypertension is defined as persisting levels of blood pressure in repeated measures equal or greater to the 95th percentile for age, height, and gender. The prevalence of arterial hypertension increased in the last decade among children and adolescents, due to the high prevalence of obesity in these age groups [7-9].

Since Brazil is a country of continental dimensions, with a population of 190 million people spread over five regions of very diverse socioeconomic and cultural characteristics, nationally representative studies are scarce. The aim of this study was to systematically review prevalence studies on hypertension in adolescents in Brazil to assess the magnitude of the problem in the country.

## Methods

### Search

This systematic review/meta-analysis sought to identify studies published from 1990 to September 2010, through searching in the following electronic databases: MEDLINE (http://www.ncbi.nlm.nih.gov/pubmed/), LILACS (http://lilacs.bvsalud.org/), SCIELO (http://www.scielo.org), WEB OF SCIENCE (http://apps.webofknowledge.com), SCOPUS (http://www.scopus.com/home.url) and ADOLEC (http://www.adolec.br/php/index.php). Last search was performed on Sep/22/2010. The references of the identified papers were also searched in order to locate studies that were not identified by the search.

The search strategy used DeCs (BIREME Health Sciences Descriptors) and MeSH (Pubmed’s Medical Subject Headings). The search was conducted with words in Portuguese and/or English (depending on the database) using three blocks of concepts: the first with terms related to age ("child" and "adolescent"), the second with terms related to hypertension ("blood pressure" and "hypertension"), and the third block with terms related to Brazil (“Brasil", "Brazil" and each state separately). The logical operator "OR" was used to match the descriptors in each block and the logical operator "AND" was used to combine the blocks together. When needed, "truncation" of the terms was performed. No search limits were applied for study design and sample size. No study was found in languages other than English, Portuguese or Spanish. Full electronic search strategy for all databases is available as Additional file 1.

### Selection of studies and data extraction

Inclusion criteria for articles were: (a) sample including adolescents (10–20 years) even if covering other age groups, as long as the data were presented separately for adolescents; (b) blood pressure measurement; (c) data collected in Brazil; (d) original research with humans; (e) studies published between 1990 and 2010. Review studies or studies with an exclusive sample of adolescents in specific health conditions (obesity, hypertension, diabetes, etc.) were excluded.

We decided to limit the year of articles in the selection because the techniques for blood pressure measurement and classification criteria for hypertension have changed since then. Moreover, the socio-demographic context was very different 20 years ago, and the prevalence at that time does not reflect the current context.

The selection of articles was based initially on the title and summary, followed by reading pre-selected articles in full. This selection was always done in pairs (ESM and LGG) and, in case of disagreement, a third person was consulted (KVB). When there was more than one publication with data from the same study, the most comprehensive article was selected.

The classification of hypertension in children and adolescents was done in accordance with the distribution curves of systolic and diastolic blood pressure by sex, age and height, observing the values corresponding to the various percentiles. Values below the 90th percentile were considered normal, as long as they were under 120/80 mmHg; values between the 90th and 95th percentiles were considered borderline (“pre-hypertension”); and values greater than or equal to the 95th percentile were considered hypertension. Also, values equal or greater than 120/80, and below the 95th percentile, were considered borderline [10].

A form was drawn up to extract data from the full text of the articles and besides hypertension prevalence the following information were extracted by a single investigator: age group, sex distribution in the sample, location and period of data collection, type of population (school or home-based), sample size, type of sample (random or not random), method of evaluation (kind of device, number of measurements, interval between measurements) and classification of hypertension (which measurements were used to). Prevalence data in this review were obtained only from studies that classified hypertension as blood pressure levels equal to or above the 95th percentile. When the authors did not describe the technique of blood pressure measurement but made reference to a guideline, it was assumed that the technique recommended in the guideline was used in the study. Authors were contacted to obtain full texts of some articles or to obtain estimates not presented in Articles.

The quality of the studies was assessed by analyzing characteristics of the studies about the design (size and type of sample); possibility of misclassification of outcome (number of measures, measures used for hypertension classification, treatment of discrepant measures) and form presentation of estimates (overall and by sex prevalence).

### Statistical analysis

Forest plots were built for the prevalence of hypertension in the overall population and by gender, when available. To obtain summary measures, we used random effects models due to the large heterogeneity of the results. To pool the prevalence measures, logit transformation were initially made to handle the distribution asymmetry. These prevalences were weighted by the inverse variance of logit [11]. Then, these combined values were converted back to prevalence. The heterogeneity between studies was assessed using I^2^ statistics [12].

Meta-regression models were adjusted in order to identify possible sources of heterogeneity among the prevalences. The variables considered for this analysis were: age, study site (region of the country), proportion of male adolescents in the study population, sample size, hypertension guideline used, type of instrument used (auscultatory or oscillometric), year of data collection, number of measurements taken and what measures were used to classify blood pressure. Firstly, univariable models were fitted including the above variables. Those with p-values equal or less than 0.20 were selected for inclusion in multivariable models [13].

Analyses were performed with STATA version 10.0 (StataCorp, 2004–2007).

PRISMA standard guideline [14] was used to guide the writing of this article. More detailed information can be found in supplementary material (Additional file 2).

## Results

### Description of included studies

**Figure 1:**
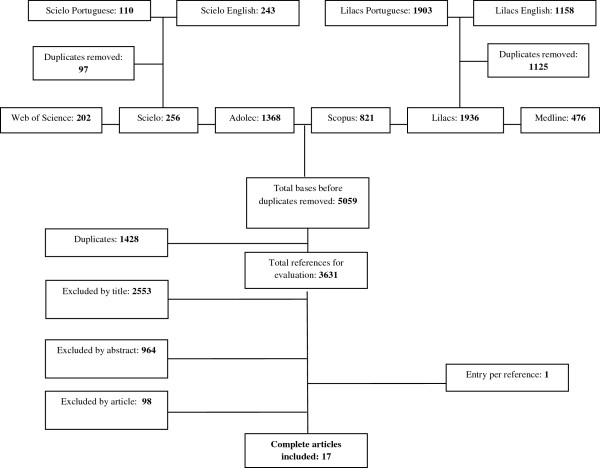
Study flowchart.

Of 3,631 studies initially identified (after duplicity exclusion), 17 were considered eligible for systematic review. No study was considered eligible through the search for theses and contacts with authors. One study was identified from the references of the selected studies (Figure 1).

The characteristics of the studies are presented in Table 1. Most studies were conducted in the Southeast (8 studies) and Northeast (6 studies) regions of Brazil. No studies from the North region were included. Most studies had their data collected from 2000 onwards, and only for two of them data was obtained between 1990 and 2000 [15,16]. Except for one home-based study [16], all the others were carried out with children/adolescents in school.

**Table 1:** Characteristics of included studies

**Reference**	**Year of data collection**	**Total N**	**Region**	**Age group (years)**	**% Male**	**Instrument**	**Number of measurements/measure used**	**Hypertension guideline used**
Brandão et al. 1996 [15]	1987	3906	SE	10-15	NI	Auscultatory	3/Only third measurement	Task Force 1987 [17]
Candido et al. 2009 [18]	2006	487	SE	10-14	48.5	Oscillometric	3	V DBHA 2006 [19]
Chaves et al. 2006 [20]	2001-2002	179	NE	12-18	50.3	Auscultatory	NI	IV DBHA 2004 [21]
Christofaro et al. 2010 [22]	2008	233	S	10-15	51.5	Oscillometric	2/Average of 2	The Fourth Report 2004 [10] V DBHA 2006 [19]
Costa et al. 1998 [16]	1995-1996	646	SE	12-19	51.4	Oscillometric	2/Average of 2	V JNC 1983 [23]
Da Silva et al. 2007 [4]	2005	674	NE	14-17	44.9	Auscultatory	2/Average of 2	The Fourth Report 2004 [10]
De Campos et al. 2010 [24]	2008	497	S	10-18	52.3	Auscultatory	2/Average of 2	The Fourth Report 2004 [10]
Gomes et al. 2009 [25]	2006	1878	NE	14-20	39.3	Auscultatory	1	V DBHA 2006 [19] Task Force 1996 [26]
Monego et al. 2006 [27]	2001-2002	2118	CO	10-14	49.7	Auscultatory	2/Second measurement	Task Force 1996 [26]
Moura et al. 2004 [28]	2000-2002	898	NE	11-17	42.3	Auscultatory	2/Assesses both measurements separately	Task Force 1996 [26] III Brazilian Consensus on Hypertension 1999 [29]
Paixão et al. 2009 [30]	2006	71	SE	11-16	46.5	Auscultatory	3/Mean of 2 last measurements (V DBHA)	V DBHA 2006 [19]
Peters et al. 2009 [31]	2006	205	SE	16-20	51.7	Auscultatory	3/Mean of 2 last measurements	V DBHA 2006 [19] The Fourth Report 2004 [10]
Rodrigues et al. 2006 [32]	2003-2005	380	SE	10-14	46.6	Auscultatory	3/Mean of 3	IV DBHA 2004 [21] Task Force 1996 [26]
Roelofs et al. 2010 [33]	2008	1002	NE	12-17	44.1	Oscillometric	3/Mean of 2 last measurements	The Fourth Report 2004 [10]
Rosa et al. 2007 [34]	2003-2004	456	SE	12-17	44.5	Oscillometric	6/Mean of 6	The Fourth Report 2004 [10] IV DBHA 2004 [21]
Silva et al. 2008 [35]	2007	704	SE	10-15	47.3	Auscultatory	3/Mean of 2 last measurements (V DBHA)	V DBHA 2006 [19]
Souza et al. 2006 [36]	2004	302	NE	12-18	31.4	Auscultatory	3	IV DBHA 2004 [21]

Regions: CO-Midwest; NE- Northeast; S-South; SE- Southeast.

NI – No information.

DBHA – Brazilian Guideline for Hypertension (Diretriz Brasileira de Hipertensão Arterial).

All studies measured blood pressure with participants in the sitting position.

All studies used the as definition of hypertension values equal to or above the 95^th^ percentile.

The sample sizes ranged from 71 to 3,906 adolescents; four of the 17 studies were conducted with samples of up to 249 individuals, five with samples of 250–499 adolescents, four with samples of 500–999 adolescents and four with samples of more than one thousand individuals. For blood pressure measurement, auscultatory method was used in 12 of the 17 studies.

There was great diversity with respect to blood pressure measurement procedures. Different cuff sizes were used in most studies, as recommended by all guidelines. However, the number of measurements taken, the interval between these measurements and the measure used for analysis varied widely among studies. Many studies did not present complete information about the blood pressure measurement method. When the measurement method was not described in detail, but the authors mentioned a reference, usually a guideline, it was considered that the study used the technique described in the reference. The guidelines referenced in the studies were: Task Force 1987 [17], IV Brazilian Guideline for Hypertension [21] and V Brazilian Guideline for Hypertension [19], The Fourth Report 2004 [10], V JNC 1983 [23], Task Force 1996 [26] and III Brazilian Consensus on Hypertension 1999 [29].

**Figure 2:**
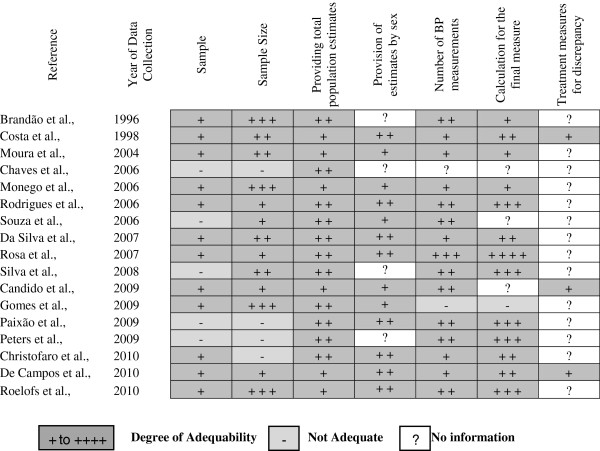
**Assessment of studies quality.** Sample: Non-random (−); Random (+). Sample Size: < 250 (−); 250–499 (+); 500–999 (+ +); ≥1000 (+ + +). General prevalences: Calculated estimated values (+); Estimates provided (+ +). Sex prevalences: Calculated estimated values (+); Estimates provided (+ +). Number of Blood Pressure (BP) Measurements: One (−); Two (+); Three (+ +); More than three (+ + +) Blood Pressure Measurement used for Classification: first (−); third one (+); mean over two (+ +); mean over the last two (+ + +); mean over three or more (+ + + +). Deal with Discrepant measurements: Yes (+).

To assess the quality of the studies, a figure was created with the analysis of the methodological characteristics of studies (Figure 2), showing the degree of appropriateness of the methods used.

Regarding the type of sample, only five studies were not random cluster samples, the majority of studies were carried out in schools, only one in household. The size of the samples was quite diverse; only four studies used samples with less than 250 participants. Several studies did not report the method of blood pressure measurement, particularly with regard to the way of dealing with discrepant measurements (if measurements with a large difference between the systolic or diastolic pressures were discarded and replaced with more homogeneous values). Most studies performed at least two blood pressure measurements and 10 out of 17 used the mean of at least two measurements to classify the blood pressure. The prevalence in the total population was extracted from eleven studies and calculated in six. For four studies it was not possible to extract or calculate prevalence by sex and in five studies they were calculated.

The prevalence of overall hypertension and the results by sex are shown in Table 2. Studies that only reported borderline blood pressure values (p90-p95) or that pooled borderline values with hypertension and presented the result as blood pressure prevalence > p90 were excluded. A wide variation of these estimates was observed. For the general population, the lowest prevalence was 2.7% [36] and the highest one was 20.1% [20]. The number of males was similar to that of females in most of the samples. In males, prevalence varied from 2.8% [27] to 24% [25], whereas in females they varied from 0% [30] to 14.2% [22]. In general, prevalence was higher in males than females, except in two studies [22,27].

**Table 2:** Prevalence of total hypertension and hypertension according to sex

**Reference**	**Total N**	**Total prevalence (%)**	**Male prevalence (%)**	**Female prevalence (%)**
	**Male N/Female N**	**(CI 95%)**	**(CI 95%)**	**(CI 95%)**
Brandão et al. 1996 [15]	3906	8.7(7.89-9.66)	NI	NI
Candido et al. 2009 [18]	487	3.3*	3.8*	2.8*
	236/251	(2.03-5.31)	(1.99-7.15)	(1.34-5.74)
Chaves et al. 2006 [20]	179	20.1	NI	NI
	90/89	(14.86-26.61)		
Christofaro et al. 2010 [22]	233	12.4	10.6	14.2
	120/113	(8.75-17.28)	(6.22-17.49)	(8.90-21.91)
Costa et al. 1998 [16]	646	8.9*	10.9	7
	332/314	(6.94-11.35)	(7.97-14.73)	(4.65-10.40)
Da Silva et al. 2007 [4]	674	7.4	10.2	5.1
	303/371	(5.65-9.64)	(7.26-14.14)	(3.27-7.86)
De Campos et al. 2010 [24]	497	12.6*	13.8	11.5
	260/237	(9.96-15.81)	(10.11-18.55)	(8.02-16.22)
Gomes et al. 2009 [25]	1878	17.3	24	13*
	738/1140	(15.65-19.07)	(21.05-27.21)	(11.17-15.08)
Monego et al. 2006 [27]	2118	2.9*	2.8*	3.1*
	1052/1066	(2.26-3.71)	(1.95-3.98)	(4.41-8.96)
Moura et al. 2004 [28]	898	10.6*	11.8*	9.6*
	380/518	(8.75-12.79)	(8.92-15.44)	(7.34-12.45)
Paixão et al. 2009 [30]	71	4.2	9	0
	33/38	(1.36-12.26)	(3.00-24.57)	(0.005-31.30)
Peters et al. 2009 [31]	205	12.2	NI	NI
	106/99	(8.37-17.42)		
Rodrigues et al. 2006 [32]	380	3.4	3.4	3.4
	177/203	(1.98-5.78)	(1.54-7.35)	(1.62-7.00)
Roelofs et al. 2010 [33]	1002	14.7*	17	12.9
	442/560	(12.64-17.03)	(13.77-20.80)	(10.36-15.94)
Rosa et al. 2007 [34]	456	4.6	5.9	3.6
	203/253	(3.02-6.95)	(3.38-10.10)	(1.90-6.74)
Silva et al. 2008 [35]	704	9.5	NI	NI
	333/371	(7.56-11.92)		
Souza et al. 2006 [36]	302	2.7	6.1	1.1*
	95/207	(1.36-5.27)	(2.73-13.09)	(0.30-3.94)

NI – No Information * Calculated values CI – Confidence Interval.

**Figure 3:**
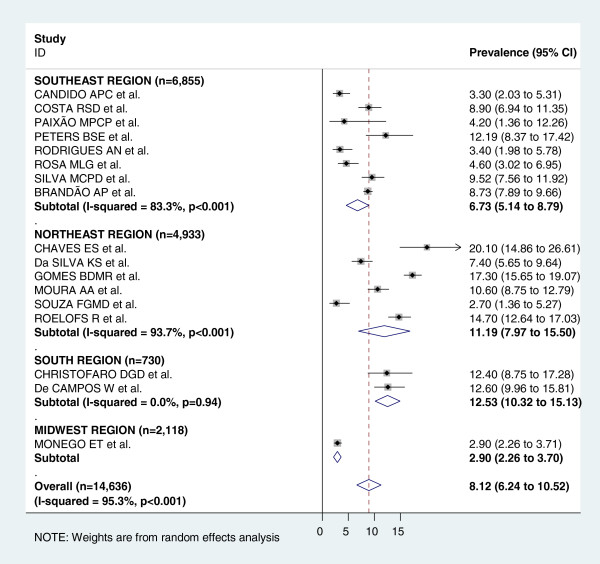
**Forest plot of hypertension prevalences in adolescents by region.** Squares represent study-specific hypertensive prevalence estimates (size of the square reflects the study-specific statistical weight, that is, the inverse of the variance); horizontal lines represent 95% Confidence Intervals; diamonds represent summary estimates of hypertension Prevalence with corresponding 95% CIs.

**Figure 4:**
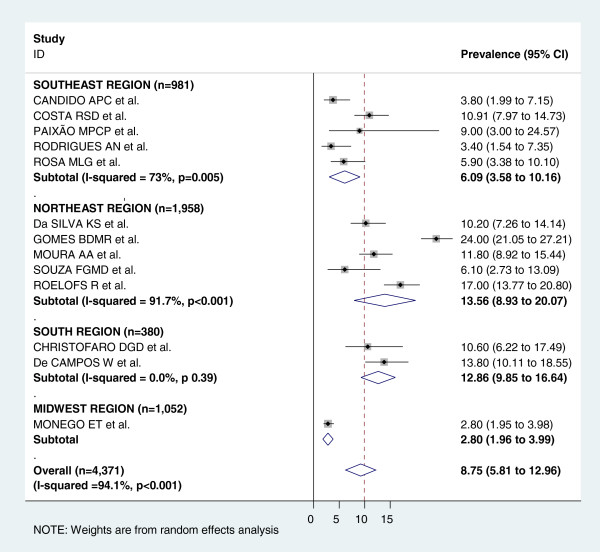
**Forest plot of hypertension prevalences in male adolescents by region.** Squares represent study-specific hypertensive prevalence estimates (size of the square reflects the study-specific statistical weight, that is, the inverse of the variance); horizontal lines represent 95% Confidence Intervals; diamonds represent summary estimates of hypertension Prevalence with corresponding 95% CIs.

**Figure 5:**
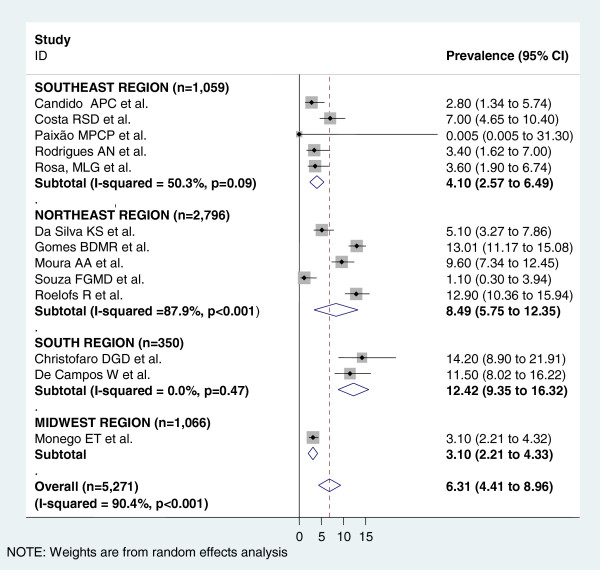
**Forest plot of hypertension prevalences in female adolescents by region.** Squares represent study-specific hypertensive prevalence estimates (size of the square reflects the study-specific statistical weight, that is, the inverse of the variance); horizontal lines represent 95% Confidence Intervals; diamonds represent summary estimates of hypertension Prevalence with corresponding 95% CIs.

Figure 3 shows the pooled measures of hypertension prevalence for the total and regional populations. For the analysis of the total population, all 17 studies were included and heterogeneity was very high (I^2^ = 95.3%). The pooled measure (prevalence) by random-effects model was 8.12%. The highest prevalence was found in the South region (12.53%) and the lowest one in the Midwest region (2.9%). Figures 4 and 5 display the forest-plot by region for male and female population. Thirteen studies were included for the combined estimate for males, with I^2^ = 94.1%. The pooled prevalence was 8.75%, with the highest prevalence found in the Northeast region (13.56%) and the lowest one in the Midwest region (2.80%). For females, thirteen studies with I^2^ = 90.4% were also included. The pooled measure was 6.31%. The highest prevalence was found in the South region (12.42%) and the lowest one in the Midwest region (3.1%).

Meta-regression analyses were conducted with the variables listed in the “selection of studies and data extraction” section. The results of meta-regression univariate analysis are available as Additional file 3. Variables included in the multivariate analysis were: age, region, sample (type and size) and number of blood pressure measurements (Table 3). The only variables that partially and inconsistently explained the observed heterogeneity were the region of the country where the study was conducted and sample. Table 3 shows the meta-regression parameters, with the Southeast region as the reference category. None of the remaining variables was associated with the variation in hypertension prevalence, both for total and stratified (by sex) populations. Publication bias was not assessed due to the large heterogeneity observed.

**Table 3:** Meta-regression parameters in total population and by sex

**Variable**	**Total population (n = 17)**		**Male (n = 13)**		**Female (n = 13)**	
	**OR (95% CI)**	**P value**	**OR (95% CI)**	**P value**	**OR (95% CI)**	**P value**
**Age**	1.15(0.96-1.36)	0.103	1.23(0.87-1.73)	0.190	-	-
Southeast	Reference		Reference		Reference	
Northeast	0.90(0.46-1.72)	0.705	1.47(0.54-3.97)	0.390	2.20(0.95-5.28)	0.071
South	3.69(1.37-9.89)	0.017	2.22(0.61-7.34)	0.159	2.89(1.01-8.22)	0.047
Midwest	0.24(0.08-0.67)	0.014	0.46(0.11-1.85)	0.228	0.64(0.18-2.16)	0.413
**Sample**						
Random	-	-	-	-	Reference	
Non random	-	-	-	-	0.11(0.02-0.66)	0.023
**Sample size**						
< 250	Reference		-	-	-	-
250-499	0.72(0.30-1.72)	0.408	-	-	-	-
500-999	1.80(0.66-4.81)	0.203	-	-	-	-
≥ 1000	2.65(0.96-7.31)	0.057	-	-	-	-
**Number of BP measurements**	1.02(0.76-1.36)	0.884	0.87(0.61-1.22)	0.361	0.91(0.65-1.26)	0.511

OR - Odds ratio.

CI = Confidence Interval.

BP - Blood pressure.

## Discussion

This systematic review showed a large variation in the hypertension prevalence estimated in studies with Brazilian adolescents. The methods of measurement varied widely, but did not explain the large heterogeneity among these findings, probably because there were no subgroups with sufficient number of studies using similar methods for comparison. There was a predominance of studies in the Southeast and Northeast regions. The small number of studies in Midwest and South regions included in this review did not allow a more detailed analysis of this finding.

Prevalence was higher in males. A study with 5,102 American schoolchildren, aged 10–19 years, also found a higher prevalence of hypertension in male adolescents (23%) when compared to female adolescents (16%) [9].

Considering the same classification criteria we used, a study in Lisbon, Portugal found a prevalence of hypertension of 34%, in 234 adolescents (43% in males and 21% in females), higher than the pooled one observed in our review. The authors argued that the findings were similar to other studies in the country [37]. On the other hand, data from NHANES, a nationally representative survey of the health and nutritional status of the noninstitutionalized population of the United States, showed hypertension prevalences in adolescents ranging from 5% (1999–2000) to 3% (2007–2008) [38]. In Hungary 10,539 adolescents with mean age 16.6 years were examined and the prevalence of hypertension was 2.53% [39]. Similar figures were found in a Chinese study that examined 88,974 scholars, 12 to 17 years, in Changsha city. Total prevalence was 3.1% (4.7% in males and 1.5% in females) [40]. A Canadian study looking for trends in cardiovascular risk and lifestyle factors in 20,719 adolescents (14- to 15-year-old) observed constant prevalences of stage I hypertension (5-6%) or stage II hypertension (2-4%) [41].

Region of the country appeared to partially explain the observed heterogeneity in prevalence among the different studies. Studies carried out in the Southeast region tend to show lower figures of prevalence when compared to South and Northeast regions. However this finding should be viewed with caution due to the small number of studies in some regions. Moreover, the number of studies with prevalence of hypertension for the total population differed from the number of studies with prevalence by gender. Studies with representative samples of all regions of Brazil comparing the prevalence of hypertension in adolescents may help identify determinants of high blood pressure since there is a large socio-cultural heterogeneity among Brazilian regions.

The high prevalences described in the studies analyzed in this meta-analysis may be overestimated since the blood pressure was measured on a single occasion in all studies. Measurements in 2 or more different occasions are operationally hard to perform in populational studies. In the USA a study found a prevalence of 19.4% of elevated blood pressure in the first assessment of schoolchildren. After 1–2 weeks only 9.5% of the students were considered hypertensive. This prevalence dropped to 4.5% in a third evaluation [9]. Other studies have also made more than one blood pressure measurement to estimate the prevalence of hypertension in children and adolescents. A cross-sectional study in 6,790 adolescents (11–17 years) in Houston found a prevalence of hypertension, at the initial screen, of 9.4%. After 3 screenings the prevalence was 3.2% [42]. Nine to ten year old students (970) in the greater Reykjavic in Iceland were recruited for a study to investigate the prevalence of hypertension. At the initial screening, 13.1% had blood pressure in the hypertensive range, 6% after the second and 3.1% following the third screening [43]. A survey with Schoolchildren (3,394 African American and 11,292 white students) aged 10–15 years in St. Paul and Minneapolis found a hypertension prevalence of 2.7% (systolic) and 2% (diastolic). After a second measurement, the prevalences were 0.8% (systolic) and 0.4% (diastolic) [44]. These results reflect the well-known phenomenon of regression to the mean, which drives the recommendation that blood pressure measurement should be taken on more than one occasion in order to establish the diagnosis of hypertension.

The lack of information about characteristics of the methods used in the studies and the great variability of measuring methods certainly hindered a more adequate analysis, as these differences could explain the observed heterogeneity. The first measure tends to be higher and using this single measure tends to overestimate the prevalence of hypertension. The average between the first and second or between the second and third measurements tends to be a more representative and suitable value, even if the measurement used is the second or the third [45].

Standardization of the procedures used in blood pressure measurement according to national and international guidelines are of utmost importance in epidemiological studies and they need to be adequately described, enabling the reader to assess the impact of methodological characteristics known to influence the accuracy of measurements.

Few studies in this meta-analysis had information on obesity, sexual maturation, or other data that could be an additional source of heterogeneity in prevalences of hypertension among adolescents besides methodological characteristics of the studies.

## Conclusion

Despite the observed heterogeneity and some risk of overestimation, the pooled prevalence found for both sexes was high and indicates that systemic arterial hypertension should be monitored in the population aged 10–20 years. Specific measures are required to prevent and control the disease and its risk factors in order to avoid future complications for young individuals, such as reduction in life expectancy for their generation [46].

## Competing interests

The authors declare that they have no conflict of interests.

## Authors’ contributions

ESM participated in the literature search, selection of articles, data extraction and analysis and writing of the manuscript. LGG participated in the selection of articles and writing of the manuscript. KVB participated in the selection of articles, data extraction and analysis and writing of the manuscript. ESFC participated in data analysis and writing of the manuscript. All authors read and approved the final manuscript.

## Pre-publication history

The pre-publication history for this paper can be accessed here:

http://www.biomedcentral.com/1471-2458/13/833/prepub

## Supplementary Material

Additional file 1 Full Search Strategy. Click here for file

Additional file 2 PRISMA Checklist. Click here for file

Additional file 3 Meta-regression: Univariate analysis. Click here for file
