# Supplementary material for: Prevalence of arterial hypertension among Brazilian adolescents: systematic review and meta-analysis
Source: BMC Public Health. 2013 Sep 11;13:833. doi: 10.1186/1471-2458-13-833 (PMC3847925; doi:10.1186/1471-2458-13-833)
Supplement: Additional file 1 — Full Search Strategy. [file 1471-2458-13-833-S1.pdf]

Additional file 1 – Full search strategy

Pubmed Search– Sep/09/2010 – http://www.ncbi.nlm.nih.gov/pubmed/

(child*[Title/Abstract] OR adolesc*[Title/Abstract] OR adolescent[MeSH] OR child[MeSH]) AND ("blood pressure"[MeSH] OR "blood pressure"[Title/Abstract] OR hypertension[MeSH] OR hypertension[Title/Abstract] OR hypert*[Title/Abstract]) AND (brazil*[Title/Abstract] OR brazil[Title/Abstract] OR brazil[MeSH] OR brasil[Title/Abstract] OR "minas gerais"[Title/Abstract] OR "são paulo"[Title/Abstract] OR "espírito santo"[Title/Abstract] OR "rio de janeiro"[Title/Abstract] OR bahia[Title/Abstract] OR pará[Title/Abstract] OR "mato grosso"[Title/Abstract] OR "mato grosso do sul"[Title/Abstract] OR goiás[Title/Abstract] OR "rio grande do sul"[Title/Abstract] OR ceará[Title/Abstract] OR pernambuco[Title/Abstract] OR "santa catarina"[Title/Abstract] OR amazonas[Title/Abstract] OR maranhão[Title/Abstract] OR tocantins[Title/Abstract] OR piauí[Title/Abstract] OR rondônia[Title/Abstract] OR roraima[Title/Abstract] OR paraná[Title/Abstract] OR acre[Title/Abstract] OR amapá[Title/Abstract] OR paraíba[Title/Abstract] OR "rio grande do norte"[Title/Abstract] OR alagoas[Title/Abstract] OR sergipe[Title/Abstract] OR "distrito federal"[Title/Abstract]) Publication Date from 1990 to 2010

Scopus Search– Sep/13/2010 – http://www.scopus.com/home/url

(TITLE-ABS-KEY(child* OR adolesc*) AND TITLE-ABS-KEY("blood pressure" OR hypertension OR hypert*) AND TITLE-ABS-KEY(brazil* OR brazil OR brasil OR "minas gerais" OR "são paulo" OR "espírito santo" OR "rio de janeiro" OR bahia OR pará OR "mato grosso" OR "mato grosso do sul" OR goiás OR "rio grande do sul" OR ceará OR pernambuco OR "santa catarina" OR amazonas OR maranhão OR tocantins OR piauí OR rondônia OR roraima OR paraná OR acre OR amapá OR paraíba OR "rio grande do norte" OR alagoas OR sergipe OR "distrito federal")) AND PUBYEAR AFT 1989

Isi Web of Science Search – Sep/13/2010 – http://apps.webofknowledge.com

TS=(child* OR adolesc*) AND TS=("blood pressure" OR hypertension OR hypert*) AND TS=(brazil* OR brazil OR brasil OR "minas gerais" OR "sao paulo" OR "espirito santo" OR "rio de janeiro" OR bahia OR para OR "mato grosso" OR "mato grosso do sul" OR goias OR "rio grande do sul" OR ceara OR pernambuco OR "santa catarina" OR amazonas OR maranhao OR tocantins OR piaui OR rondonia OR roraima OR parana OR acre OR amapa OR paraiba OR "rio grande do norte" OR alagoas OR sergipe OR "distrito federal") Timespan=1990-2010. Databases=SCI-EXPANDED, SSCI, A&HCI.

Adolec Search – Sep/13/2010 – http://www.adolec.br/php/index.php

criança$ OR adolesc$ [Palavras] and "pressão arterial" OR hipertensão OR hipert$ [Palavras] and brasil$ OR brasil OR "minas gerais" OR "são paulo" OR "espírito santo" OR "rio de janeiro" OR bahia OR pará OR "mato grosso" OR "mato grosso do sul" OR goiás OR "rio grande do sul" OR ceará OR pernambuco OR "santa catarina" OR amazonas OR maranhão OR tocantins OR piauí OR rondônia OR roraima OR paraná OR acre OR amapá OR paraíba OR "rio grande do norte" OR alagoas OR sergipe OR "distrito federal" [Palavras] "data limit option not available"

Lilacs – Sep/13/2010 – http://lilacs.bvsalud.org/

English Search: child$ OR adolesc$ [Words] and "blood pressure" OR hypertension OR hypert$ [Words] and brazil$ OR brazil OR brasil OR "minas gerais" OR "são paulo" OR "espírito santo" OR "rio de janeiro" OR bahia OR pará OR "mato grosso" OR "mato grosso do sul" OR goiás OR "rio grande do sul" OR ceará OR pernambuco OR "santa catarina" OR amazonas OR maranhão OR tocantins OR piauí OR rondônia OR roraima OR paraná OR acre OR amapá OR paraíba OR "rio grande do norte" OR alagoas OR sergipe OR "distrito federal" [Words] "data limit option not available"

Portuguese Search: criança$ OR adolesc$ [Palavras] and "pressão arterial" OR hipertensão OR hipert$ [Palavras] and brasil$ OR brasil OR "minas gerais" OR "são paulo" OR "espírito santo" OR "rio de janeiro" OR bahia OR pará OR "mato grosso" OR "mato grosso do sul" OR goiás OR "rio grande do sul" OR ceará OR pernambuco OR "santa catarina" OR amazonas OR maranhão OR tocantins OR piauí OR rondônia OR roraima OR paraná OR acre OR amapá OR paraíba OR "rio grande do norte" OR alagoas OR sergipe OR "distrito federal" [Palavras] "data limit option not available"

Scielo – Sep/22/2010 – http://www.scielo.org

Portuguese Search: SciELO.org > Pesquisa > (criança$ OR adolesc$) and ("pressão arterial" OR hipertensão OR hipert$) and (brasil$ OR brasil OR "minas gerais" OR "são paulo" OR "espírito santo" OR "rio de janeiro" OR bahia OR pará OR "mato grosso" OR "mato grosso do sul" OR goiás OR "rio grande do sul" OR ceará OR pernambuco OR "santa catarina" OR amazonas OR maranhão OR tocantins OR piauí OR rondônia OR roraima OR paraná OR acre OR amapá OR paraíba OR "rio grande do norte" OR alagoas OR sergipe OR "distrito federal") "data limit option not available"

English Search: SciELO.org > Search > (child$ OR adolesc$) and ("blood pressure" OR hypertension OR hypert$) and (brazil$ OR brazil OR brasil OR "minas gerais" OR "são paulo" OR "espírito santo" OR "rio de janeiro" OR bahia OR pará OR "mato grosso" OR "mato grosso do sul" OR goiás OR "rio grande do sul" OR ceará OR pernambuco OR "santa catarina" OR amazonas OR maranhão OR tocantins OR piauí OR rondônia OR roraima OR paraná OR acre OR amapá OR paraíba OR "rio grande do norte" OR alagoas OR sergipe OR "distrito federal") "data limit option not available"
