# Supplementary material for: Prevalence of arterial hypertension among Brazilian adolescents: systematic review and meta-analysis
Source: BMC Public Health. 2013 Sep 11;13:833. doi: 10.1186/1471-2458-13-833 (PMC3847925; doi:10.1186/1471-2458-13-833)
Supplement: Additional file 2 — PRISMA Checklist. [file 1471-2458-13-833-S2.pdf]

Section/Topic	#	Checklist Item	Reported on Page #
**TITLE**			
Title	1	Identify the report as a systematic review, meta-analysis, or both.	1
**ABSTRACT**			
Structured summary	2	Provide a structured summary including, as applicable: background; objectives; data sources; study eligibility criteria, participants, and interventions; study appraisal and synthesis methods; results; limitations; conclusions and implications of key findings; systematic review registration number.	2 (Paragraphs 1-4)
**INTRODUCTION**			
Rationale	3	Describe the rationale for the review in the context of what is already known.	3 (Paragraphs 1-4)
Objectives	4	Provide an explicit statement of questions being addressed with reference to participants, interventions, comparisons, outcomes, and study design (PICOS).	3 (5th paragraph)
**METHODS**			
Protocol and Registration	5	Indicate if a review protocol exists, if and where it can be accessed (e.g., Web address), and, if available, provide registration information including registration number.	Not Applicable
Eligibility criteria	6	Specify study characteristics (e.g., PICOS, length of follow-up) and report characteristics (e.g., years considered, language, publication status) used as criteria for eligibility, giving rationale.	4 (3rd paragraph)<br>5 (1st paragraph)
Information sources	7	Describe all information sources (e.g., databases with dates of coverage, contact with study authors to identify additional studies) in the search and date last searched.	4 (1st paragraph)
Search	8	Present full electronic search strategy for at least one database, including any limits used, such that it could be repeated.	4 (2nd paragraph) Additional file 1
Study selection	9	State the process for selecting studies (i.e., screening eligibility, included in systematic review, and, if applicable, included in the meta-analysis.	5 (Paragraphs 2-3)
Data collection process	10	Describe method of data extraction from reports (e.g., piloted forms, independently, in duplicate) and any processes for obtaining and confirming data from investigators.	5 (4th paragraph)
Data items	11	List and define all variables for which data were sought (e.g., PICOS, funding sources) and any assumptions and simplifications made.	5 (4th paragraph)
Risk of bias in individual studies	12	Describe methods used for assessing risk of bias in individual studies (including specification of whether this was done at the study or outcome level), and how this information is to be used in any data synthesis.	6 (1st paragraph)
Summary measures	13	State the principal summary measures (e.g., risk ratio, difference in means).	6 (2nd paragraph)
Synthesis of results	14	Describe the methods of handling data and combining results of studies, if done, including measures of consistency (e.g., I^2) for each meta-analysis.	6 (2nd paragraph)
Risk of bias across studies	15	Specify any assessment of risk of bias that may affect the cumulative evidence (e.g., publication bias, selective reporting within studies).	Not applicable
Additional analysis	16	Describe methods of additional analysis (e.g., sensitivity or subgroup analysis, meta-regression), if done, indicating which were pre-specified.	6 (3rd paragraph)

**RESULTS**			
Study selection	17	Give number of studies screened, assessed for eligibility, and included in the review, with reasons for exclusions at each stage, ideally with a flow diagram.	7 (1st paragraph) and Figure 1
Study characteristics	18	For each study, present characteristics for which data were extracted (e.g., study size, PICOS, follow-up period) and provide the citations.	7 (Paragraphs 2-4) Table 1
Risk of bias within studies	19	Present data on risk of bias of each study and, if available, any outcome-level assessment (see item 12).	8 (Paragraphs 1-2) Figure 2
Results of individual studies	20	For all outcomes considered (benefits or harms), present, for each study: (a) simple summary data for each intervention group and (b) effect estimates and confidence intervals, ideally with a forest plot.	8 (3rd paragraph) Table 2
Synthesis of results	21	Present results of each meta-analysis done, including confidence intervals and measures of consistency.	8 (4th paragraph) 9 (1st paragraph) Figure 3 Figure 4 Figure 5
Risk of bias across studies	22	Presents results of any assessment of risk of bias across studies (see item 15).	9 (2nd paragraph)
Additional analysis	23	Give results of additional analysis, if done (e.g., sensitivity or subgroup analysis, meta-regression [see item 16]).	9 (2ndparagraph) Table 3 Additional file 3
**DISCUSSION**			
Summary of evidence	24	Summarize the main findings including the strength of evidence for each main outcome; consider their relevance to key groups (e.g., health care providers, users, and police makers).	9 (Paragraphs 3-4) 10 (Paragraphs 2-3) 11 (Paragraphs 3-4)
Limitations	25	Discuss limitations at study and outcome level (e.g., risk of bias), and at review level (e.g., incomplete retrieval of identified research, reporting bias).	10 (3rd paragraph) 11 (Paragraphs 2 and 4)
Conclusions	26	Provide a general interpretation of the results in the context of other evidence, and implications for future research.	11 (5th paragraph) 12 (1st paragraph)
**FUNDING**			
Funding	27	Describe search of funding for the systematic review and other support (e.g., supply of data; role of funders for the systematic review).	Not applicable
