# Supplementary material for: Prevalence of arterial hypertension among Brazilian adolescents: systematic review and meta-analysis
Source: BMC Public Health. 2013 Sep 11;13:833. doi: 10.1186/1471-2458-13-833 (PMC3847925; doi:10.1186/1471-2458-13-833)
Supplement: Additional file 3 — Meta-regression: Univariate analysis. [file 1471-2458-13-833-S3.pdf]

Meta-regression: Univariate Analysis

Variable	Total Population		Male		Female	
	Coeficient	p	Coeficient	p	Coeficient	p
**Age**	0.132	0.186	0.272	0.052	0.077	0.645
**Region**						
Southeast	Reference					
Northeast	0.574	0.110	0.883	0.031	0.754	0.126
South	0.726	0.159	0.761	0.127	1.262	0.053
Midwest	-0.839	0.212	-0.819	0.180	-0.254	0.726
**Sample**						
Random	Reference					
Non random	0.095	0.814	-0.237	0.726	-2.006	0.043
**Instrument**						
Auscultatory	Reference					
Oscilometric	-0.042	0.915	0.016	0.972	0.250	0.615
**Sample**						
School	Reference					
Household	0.121	0.871	0.261	0.742	0.159	0.856
**Sample Size**						
<250	Reference					
250-499	-1.029	0.040	-0.540	0.460	-1.104	0.224
500-999	-0.324	0.497	0.110	0.885	-0.462	0.611
≥ 1000	-0.302	0.523	0.132	0.862	-0.290	0.747
**Year of Data Collection**	0.011	0.726	0.050	0.400	0.058	0.288
**Number of BP Measurements**	-0.241	0.135	-0.245	0.181	-0.287	0.144
**Interval between BP Measurements**	0.016	0.739	0.022	0.707	0.020	0.781
**Measurement**						
Used first measure	Reference					
Any other	-0.047	0.903	-0.218	0.714	-0.261	0.682
**Percentage of males**	0.032	0.383	-	-	-	-

BP - Blood pressure
